# How Supraphysiological Oxygen Levels in Standard Cell Culture Affect Oxygen-Consuming Reactions

**DOI:** 10.1155/2018/8238459

**Published:** 2018-09-30

**Authors:** Jeffrey A. Stuart, Joao Fonseca, Fereshteh Moradi, Cassandra Cunningham, Bishoy Seliman, Cydney R. Worsfold, Sarah Dolan, John Abando, Lucas A. Maddalena

**Affiliations:** ^1^Department of Biological Sciences, Brock University, St. Catharines, ON, Canada L2S 3A1; ^2^MRC Cancer Research Centre, University of Cambridge, Hutchison/MRC Research Centre, Box 197, Cambridge Biomedical Campus, Cambridge CB2 0XZ, UK

## Abstract

Most mammalian tissue cells experience oxygen partial pressures *in vivo* equivalent to 1–6% O_2_ (i.e., physioxia). In standard cell culture, however, headspace O_2_ levels are usually not actively regulated and under these conditions are ~18%. This drives hyperoxia in cell culture media that can affect a wide variety of cellular activities and may compromise the ability of *in vitro* models to reproduce *in vivo* biology. Here, we review and discuss some specific O_2_-consuming organelles and enzymes, including mitochondria, NADPH oxidases, the transplasma membrane redox system, nitric oxide synthases, xanthine oxidase, and monoamine oxidase with respect to their sensitivities to O_2_ levels. Many of these produce reactive oxygen and/or nitrogen species (ROS/RNS) as either primary end products or byproducts and are acutely sensitive to O_2_ levels in the range from 1% to 18%. Interestingly, many of them are also transcriptional targets of hypoxia-inducible factors (HIFs) and chronic cell growth at physioxia versus 18% O_2_ may alter their expression. Aquaporins, which facilitate hydrogen peroxide diffusion into and out of cells, are also regulated by HIFs, indicating that O_2_ levels may affect intercellular communication via hydrogen peroxide. The O_2_ sensitivities of these important activities emphasize the importance of maintaining physioxia in culture.

## 1. Introduction

Mammalian cells are typically cultured under hyperoxic conditions. While most cells experience oxygen levels of 1–6% *in vivo* (physioxia; Table 1), almost all mammalian cell culture is done in humidified atmospheric air at 37°C with CO_2_ added to 5%. Although the headspace O_2_ level is usually not measured, it is 18-19% under these conditions due to displacement of O_2_ by water vapour and CO_2_. When O_2_ levels used in cell culture experiments are measured and reported, these are virtually always those of headspace gas and not media. Particularly in metabolically active cells growing in high density, the pericellular media O_2_ levels that cells experience may be substantially lower than headspace O_2_ levels [1–3], since O_2_ is continually removed from media by mitochondrial respiration and by other O_2_-consuming cellular activities.

O_2_ participates in many metabolic reactions, some of which are sensitive to O_2_ levels in the range between physioxia and 18%. Therefore, mammalian cells should be cultured in physiologically relevant O_2_ levels. Where this is not done, it is important to be aware of the potential consequences on cellular functions. One potential consequence of elevated O_2_ in cell culture media is increased cellular production of reactive oxygen (ROS) and nitrogen (RNS) species. This probably contributes to the observed effects of high O_2_ levels on cell senescence, differentiation, and apoptosis, amongst a wide range of other less well-characterized effects ([4–6]; Fehrer et al. 2007).

There is a growing appreciation of the role of O_2_ levels in cell biology (e.g., [7, 8]). However, there is a limited mechanistic understanding of how supraphysiological O_2_ levels influence specific O_2_-dependent processes. In this review, we consider the O_2_ sensitivity of some of the important O_2_-consuming organelles and enzymes in mammalian cells in the range between physioxia and 18% O_2_ (standard cell culture conditions). We use the Michaelis-Menten constant (K_m_(O_2_)) for each O_2_-consuming enzyme (where this value is available) as a convenient way to compare O_2_ sensitivity across a broad range of different organelles and enzymes. All O_2_ and K_m_(O_2_) values from published works have been converted to % O_2_ at 37°C, since % O_2_ is the unit of measurement used in most descriptions of cell culture experiments. In general, higher K_m_(O_2_) values are indicative of enzymes that will be more sensitive to the differences between *in vivo* and *in vitro* O_2_ levels. We address the physiological role(s) of the metabolite(s) produced from these O_2_-consuming reactions and the O_2_ sensitivity over the range from physiological to 18% O_2_ of that reaction. We further summarize the interesting observation that many of the O_2_-consuming and ROS/RNS-producing enzymes are positively regulated by hypoxia, in some instances specifically by hypoxia-inducible factor-1 (HIF-1).

### 1.1. Oxygen Limitation of Mitochondrial Respiration in Cell Culture

An important goal of maintaining higher O_2_ levels in culture is to ensure that mitochondrial respiration is not limited by O_2_ availability. Some of the most comprehensive and physiologically relevant data on the O_2_ levels required to sustain maximal mitochondrial respiration rates have been provided by Hoffmann et al. (2009), who measured these values for isolated liver mitochondria while systematically varying O_2_ concentrations. State 4 respiration of complex I or complex II substrates (glutamate/malate or succinate, respectively) or of palmitoyl carnitine is near maximal at ~1% O_2_ (Hoffman 2009). Marcinek et al. (2003) showed that respiration in skeletal muscle is not O_2_ limited until O_2_ falls below ~0.5%, which is similar to the observation by Gnaiger (2001) for isolated rat liver mitochondria. To understand how this relates to O_2_ sufficiency in cell culture, we can compare these values to the levels of O_2_ present in media immediately outside of cells (pericellular O_2_) or within the cytosol (Table 2).

Pericellular O_2_ values for adherent cell monolayers can be measured in real time using a variety of approaches, including Luxcel and Seahorse platforms (Agilent, USA). We use PreSens OxyDish (PreSens Precision Sensing GMBH, Germany) which has O_2_-sensitive fluorescent probes impregnated into the tissue culture dish plastic. For most cell lines seeded at typical densities and maintained in a 5% CO_2_ incubator at 37°C without O_2_ control, headspace O_2_~18% and pericellular O_2_ levels are close to this [3]. This is far in excess of what is needed to support maximal mitochondrial respiration rates. When headspace O_2_ is maintained at more physiologically relevant levels, however, pericellular and intracellular O_2_ levels may become significantly lower, particularly if media changes and/or mixing are infrequent.

Intracellular O_2_ levels can be measured in cultured cells using a variety of fluorescent probes (Zhdanov et al. 2012; [9]). Measurements using these tools show that, at the higher levels of O_2_ (~20%) typical of standard cell culture, intracellular O_2_ levels range from 14–17% depending upon cell type, medium composition (specifically whether fuel source promotes reliance on oxidative phosphorylation), and seeding density (Table 2). Conditions promoting faster rates of O_2_ consumption lower these values, but they are generally hyperoxic regardless. At more physiologically relevant headspace O_2_ levels of 5–9%, intracellular O_2_ levels under typical culture conditions range from 0.5% to 5%. Thus, under standard cell culture conditions, intracellular O_2_ levels are typically at least 10 times higher than what is required to sustain maximal mitochondrial respiration rates based on the V_max_ values reported by Hoffman et al. (2009) and others. On the other hand, cell culture at 5-6% headspace O_2_ results in intracellular O_2_ levels that may be low enough to limit respiration when cells are at high density or growing in respiration-promoting media.

Although a higher than physiological headspace O_2_ level in tissue culture helps to ensure that mitochondrial respiration is not oxygen limited, it may have the unintended consequence of stimulating the production of ROS and RNS from various enzymes that are widely expressed in common cell lines. Increases in ROS/RNS with concomitant effects on redox-sensitive signaling events and potentially oxidative macromolecular damage should be expected under these conditions, and indeed, these are observed. It is therefore important to understand the relationship between oxygen levels and the rates of ROS/RNS production in cultured cells. In mammalian cells, ROS/RNS are produced by a wide range of organelles and enzymes, including mitochondria, NADPH oxidase (Nox), nitric oxide synthase (NOS), monoamine oxidase (MAO), xanthine oxidase/oxidoreductase (XO/XOR), lipoxygenase (LOX), cyclooxygenase (COX), heme oxygenase (HOX), and the transplasma membrane redox system (tPMRS). Here, we discuss how the hyperoxia of standard cell culture is expected to affect the activities and/or expression of all these organelles and enzymes. Our list of oxygen-dependent enzymes is not exhaustive, and we have omitted some oxygen-metabolizing enzymes for which we could not readily find data regarding oxygen sensitivity of reaction rates.

### 1.2. Oxygen Concentration and Mitochondrial ROS Production

**Figure 1:**
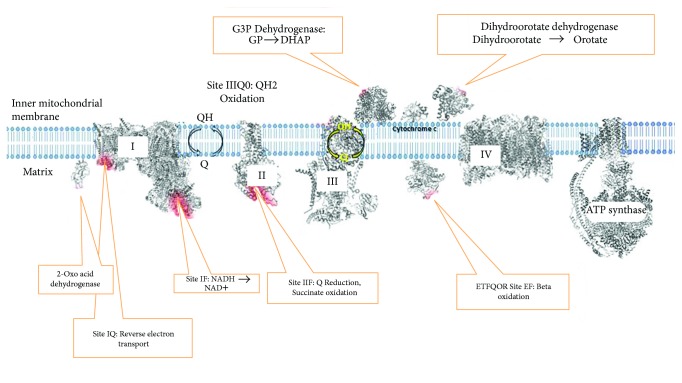
Sites of H_2_O_2_ production in the mitochondrial electron transport chain. These include 2-oxoacid dehydrogenase, complex I (site I_F_), complex I (site I_Q_), complex II (site II_F_), complex III (site III_Q0_), the ETFQOR system (site E_F_), glycerol 3-phosphate dehydrogenase (site G_q_), and dihydroorotate dehydrogenase (site D_q_). Complex I: O00217 (*Homo sapiens*); complex II: Q0QF01 (*Sus scrofa*); complex III: P23004 (*Bos taurus*); complex IV: P68530 (*Bos taurus*); ATP synthase O00217 (*Bos taurus*); cytochrome c: P99999 (*Homo sapiens*); glycerol 3-phosphate: P04406 (*Homo sapiens*); dihydroorotate dehydrogenase: ID3H (*Homo sapiens*); 2-oxoacid dehydrogenase: 2DNE (*Homo sapiens*); ETFQOR: 2GMH (*Sus scrofa).* Data based on Wong et al. [10]. Structures retrieved from https://www.rcsb.org/.

Superoxide/H_2_O_2_ production as a byproduct of oxidative phosphorylation has been well studied in isolated mitochondria, and many sites of production, albeit under generally nonphysiological conditions, have been identified (Figure 1). The specific sites of mitochondrial superoxide production have been reviewed recently (e.g., [10]) and will not be detailed here. Superoxide produced at various sites within mitochondria is released into either the matrix or the IMS side of the inner membrane. H_2_O_2_ arising from superoxide within the mitochondrial matrix can diffuse out of the matrix.

Although mitochondria are often stated to be responsible for the majority of cellular ROS production, this has not been demonstrated [11] and indeed seems unlikely to be universally true given that the total cell volume occupied by mitochondria varies from a few percent in low-metabolic rate cells to as much as 30% in cardiomyocytes [12]. Similarly, the relative levels of other ROS and RNS producers like Nox and NOS vary greatly between cell types and physiological condition. Therefore, while it may be true that mitochondria are the most important sites of ROS production in *some* cell types, they may not be in others. Nonetheless, it is important to consider the sensitivity of mitochondrial ROS production to the oxygen levels prevailing in cells in culture.

Hoffman et al. (2007; 2009) provided detailed measurements and calculations of H_2_O_2_ production (originating as superoxide) from isolated liver mitochondria respiring in state 4 on various substrates at 37°C (Table 3). Measurements were made over a range of O_2_ levels, with and without various respiratory poisons, allowing the calculation of K_m_(O_2_) values for H_2_O_2_ production associated with different respiratory substrates and ETC sites. Notably, of the sites contributing to mitochondrial H_2_O_2_ production in cultured cells, which is likely primarily the complex I FMN site and the complex III Q_o_ site, both are saturated at the same low O_2_ levels as respiration. Note that ETFQOR is probably not a major contributor to mitochondrial H_2_O_2_ production under standard cell culture conditions because fatty acids are not included as fuels in most commercial media. The physiological relevance of complex I backflow is established only in ischemia/reperfusion [13], and these would be expected to occur in normal cell culture only as hypoxia-hyperoxia transitions during media changes or passaging. Therefore, based on the above observations, the rate of mitochondrial ROS production is likely not O_2_-limited under most standard cell culture conditions or at headspace O_2_ levels as low as 5%, providing that intracellular O_2_ remains above 0.5%. Thus, little difference is expected in mitochondrial ROS production between 5% (physioxia) and 18% (standard cell culture) O_2_.

### 1.3. NADPH Oxidases

There are seven members of the family of nicotinamide adenine dinucleotide phosphate (NADPH) oxidases (Nox1–5 and Duox 1 and 2), several of which (e.g., Nox1, 2, and 4) are quite widely distributed amongst mammalian tissues and cell lines [14]. Noxs are multisubunit membrane-spanning enzymes that transport electrons from NAD(P)H across biological membranes [15]. Although the Nox enzymes localize to a variety of cellular membranes, all can be found in the plasma membrane, where they transport electrons to external O_2_, thus leading to the production of superoxide in the extracellular space [14]. Superoxide produced outside the cell and dismuted to H_2_O_2_ can diffuse back into the cell of origin or into neighbouring cells.

Importantly, recent work indicates that Nox4 differs from other Nox isoforms in several important ways. Firstly, Nox4 localizes (at least in some cells and/or some physiological conditions) to mitochondria [16] where its activity appears to interact with respiratory complex I and ATP [17, 18]. Nox4 activity is not dependent upon interactions with accessory subunits. Furthermore, Nox4 preferentially produces H_2_O_2_ rather than superoxide [19]. The rate of Nox4 H_2_O_2_ production is also very sensitive to O_2_ levels in the range between physioxia and 18%.

**Figure 2:**
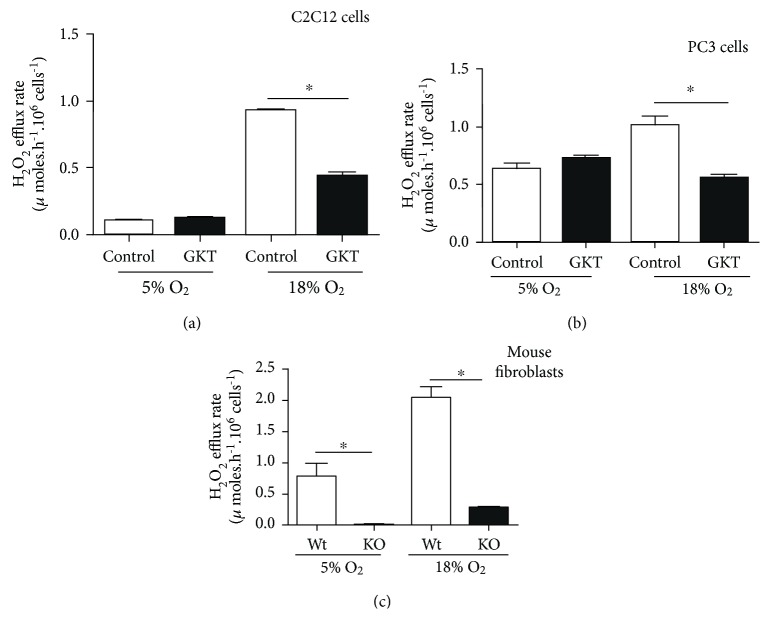
Cells produce more H_2_O_2_ at 18% versus 5% O_2_, but this is prevented or ameliorated by selective NADPH oxidase 1/4 inhibitor GKT137831. (a) C2C12 mouse myoblasts, (b) PC-3 human prostate cancer cells, and (c) wild-type and NOX1/2/4 triple knockout mouse dermal fibroblast cell lines were grown at 18% O_2_ in a humidified 37°C CO_2_ incubator, at 5%, and assayed at either 5% or 18% O_2_ as in Maddalena et al. [3]. H_2_O_2_ efflux from cells was measured using Amplex Red reagent (10-acetyl-3,7-dihydroxyphenoxazine). Krebs-Ringer buffer (KRB) was used for the assays which consisted of 135 mM NaCl, 5 mM KCl, 1 mM MgSO_4_, 0.4 mM K_2_HPO_4_, 20 mM HEPES, 5.5 mM glucose, and 10% fetal bovine serum. During the experiment, cells were incubated in KRB-contained 50 mM Amplex Red reagent and 0.1 units/mL horseradish peroxidase enzyme in the presence of 5 *μ*M GKT 137831 or vehicle control. Data were analysed using two-tailed *t*-tests. Bars represent the mean ± SEM from at least five independent experiments. ^∗^*p* < 0.05.

There is surprisingly little K_m_(O_2_) data for any of the Nox isoforms. Furthermore, there is some doubt regarding the validity of some assays of Nox activity using isolated membrane fractions [20, 21]. Nonetheless, the available data suggest that the activities of Nox1, Nox2, and Nox4 are all sensitive to O_2_ levels in the range from 5% to 18% (Table 4). Nox4, with a K_m_(O_2_) value~18%, is particularly sensitive over this range; Nox4 activity triples between 3% and 12% O_2_ [19]. Direct measurements with cultured PC3 and C2C12 cells indicate a substantial contribution of Nox1 and/or Nox4 to H_2_O_2_ production in live cells (measured as Amplex Red oxidation), particularly at 18% O_2_, since this value is strongly inhibited by GKT138731 (a Nox1/4 inhibitor; Figure 2). Similarly, in Nox1/2/4 triple knockout mouse dermal fibroblasts, H_2_O_2_ production at 18% O_2_ is only about 1/10 to that in wild-type fibroblasts, and, at 5% O_2_, H_2_O_2_ production is undetectable (Figure 2). Thus, although somewhat limited, available evidence indicates that Nox isoforms produce H_2_O_2_ at rates that are strongly dependent upon O_2_ levels. This probably underlies observations such as the Nox4 contribution to cellular senescence in primary cell lines, since these studies have been performed at 18% O_2_. Whether Nox4 plays the same roles *in vivo*, where O_2_ levels are several times lower, is not clear but must be considered.

### 1.4. Transplasma Membrane Redox System

The transplasma membrane electron transfer (tPMET) system is a ubiquitous system for transferring electrons from cytosolic NAD(P)H outside of the cell, similar to the Noxs [22]. The core components of the tPMET system are the NAD(P)H-quinone oxidoreductase (NQO1), NADH-cytochrome b5 reductase (CytB5red), coenzyme Q_10_, and the ecto-NADH oxidase disulfide thiol exchanger (ENox). Both NQO1 and CytB5red are distributed in multiple intracellular localizations, including in association with the plasma membrane.

The tPMET system makes significant contributions to cellular O_2_ consumption in many common cell lines (e.g., Jurkat, RAW264.7, and pancreatic beta cells; see [23, 24]). The tPMET system is highly upregulated in respiration-deficient (*ρ*^0^) cells, which accumulate coenzyme Q_10_ in the plasma membrane [25]. In *ρ*^0^ cells, the tPMRS may be the predominant site of O_2_ consumption [24].

A variety of extracellular terminal electron acceptors are possible, including O_2_, which can undergo single-electron reduction to produce superoxide. Purified mammalian CytB5red produces superoxide directly [26], though its overexpression has beneficial effects in some specific contexts [27, 28]. NQO1 is associated with an intracellular antioxidant function (e.g., [29]) but contributes to reduction of extracellular electron acceptors, presumably including O_2_, in pancreatic beta cells [23]. ENox proteins include several isoforms—the age-related arNox may be the most relevant in terms of ROS production in cell culture. This isoform produces superoxide and becomes more highly expressed in the tissues of aged mammals and in late-passage or senescent cells in culture [30].

The tPMET system has only been measured under standard cell culture O_2_ (18%), and we are not aware of any data regarding the sensitivity of superoxide production to O_2_ concentration for the system as a whole or for individual components of the system. Nonetheless, consideration should be given to the possibility that the tPMRS is O_2_ sensitive in the 5–18% range and that O_2_-dependent changes in its activity could affect cell physiology.

### 1.5. Role of Aquaporins in Transmembrane H_2_O_2_ and Gas Diffusion

The tPMRS and all Nox isoforms (a portion of all Nox isoforms localizes to the plasma membrane) can produce either superoxide or H_2_O_2_ on the extracellular side of the plasma membrane. Here, these ROS may react with membranes or membrane-bound proteins facing the extracellular space on the originating cell or on neighbouring cells. Alternatively, H_2_O_2_, either directly produced or resulting from superoxide dismutation, may cross cell membranes to exert intracellular effects. The H_2_O_2_ permeability of phospholipid bilayers is limited; indeed, H_2_O_2_ is less membrane permeant than H_2_O [31]. However, H_2_O_2_ rapidly equilibrates across cell membranes via aquaporins (AQPs). Structural studies suggest that all water-transporting AQPs may facilitate some degree of H_2_O_2_ movement across cellular membranes [32]. However, empirical data is less equivocal, suggesting that hAQP3 and hAQP8 are particularly good H_2_O_2_ transporters [31].

Interestingly, O_2_ levels modulate the expression of several AQPs. AQP1 and AQP3 mRNA and protein levels are both increased at 1% versus 20% O_2_ ([33]; Hoogewijs et al. 2016), though AQP5 and AQP9 expressions are reduced at lower O_2_ levels ([34]; Castro-Parodi et al. 2013). There is also some evidence that hypoxia can affect AQP8 permeability directly via posttranslational modifications [35]. Recent studies ([36]; Zwiazek et al. 2017) also suggest that some AQP isoforms facilitate the transport of O_2_ and therefore may facilitate O_2_ uptake by cells at low O_2_ levels. There is also strong evidence that AQP1 and AQP4 can transport other important ROS/RNS, such as NO [37, 38]. Taken together, these observations indicate that media O_2_ levels are likely to modulate the H_2_O_2_, NO, and O_2_ permeability of cell membranes via their effects on AQP expression and/or activity. This makes it more difficult to understand the detailed kinetics of how ROS/RNS and gases traverse cellular membranes *in vivo* when measurements are done *in vitro* under nonphysiological O_2_ conditions.

### 1.6. Nitric Oxide Synthases

NOS are a family of enzymes responsible for the production of NO and L-citrulline from L-arginine and O_2_ [39, 40]. Three isoforms of NOS have been identified: endothelial NOS (eNOS), neuronal NOS (nNOS), and inducible NOS (iNOS) [41]. These three NOS isoforms share approximately 50% amino acid identity [41], and all three are widely expressed in mammalian tissues and cell lines. Under some conditions, including transient anoxia/reoxygenation, NOS catalyzes an “uncoupled” reaction producing superoxide instead of NO (Stuehr et al. 1991; [42, 43]; reviewed in [44]).

The first detailed study of the O_2_ sensitivity of the NOS reaction was by Rengasamy and Johns [45] who calculated a K_m_(O_2_) value of 0.63 to 2.31% O_2_ for NOS isolated from bovine brain and aortic endothelium as well as RAW 264.7 mouse macrophages. Subsequently, K_m_(O_2_) values have been published for various tissues and cell lines, as well as for specific purified NOS isoforms (Table 4). Although these values vary widely, presumably due to differences in experimental conditions, O_2_ affinities of nNOS and iNOS are found relatively consistently to be within a range that is sensitive to changes in O_2_ levels between physioxia and 18% O_2_. Cell lines with high levels of these two isoforms will therefore produce more NO under standard cell culture conditions than *in vivo*. Cell culture models of ischemia/reperfusion injury typically employ a period of near anoxia followed by return to “normoxia” where the latter is 18% O_2_. NOS activity can be uncoupled under these conditions, and the rate of superoxide production upon reoxygenation at 18% O_2_ likely exceeds that which would occur upon a return to physioxia *in vivo*.

NO can also be produced by the reduction of nitrite catalyzed by several enzymes including XO/XOR [46]. This reaction is promoted at lower O_2_ levels and makes it difficult to predict the effect of O_2_ levels on NO production rates. Thus, the relationship between cellular NO synthesis rates and O_2_ levels will depend on the relative abundance of different NOS isoforms, which have different sensitivities to O_2_.

In addition to its effects on NO synthesis, O_2_ levels influence NO metabolism ([47, 48]; reviewed in [49]). NO is metabolized in cells by poorly characterized pathways. However, it is known that the rate of NO metabolism by cells is faster at higher O_2_ levels [50]. Thus, higher O_2_ levels will affect the rate of NO production from nNOS and iNOS while simultaneously increasing the rate of their metabolism. Again, it is difficult to predict how this will affect steady-state NO levels in all cells. In activated RAW 264.7 cells in culture, the maximum rates of NO production were observed at 8% O_2_ [50], though the relationship between O_2_ and NO levels in other cell types is unknown. The O_2_-dependent rate of NO metabolism will in turn affect its diffusional distance and therefore alter the subset of proteins modified in the cell of origin or neighbouring cells. NO participates in many regulatory posttranslational modifications of key proteins, including those driving epigenetic modifications. Given these diverse influences of NO on cellular functions and the effects of O_2_ levels on synthesis, diffusion distance, and metabolism of NO, there is clear potential to generate nonphysiologically relevant results at nonphysiological O_2_ levels.

### 1.7. Other Oxidases

XO and MAO are two widely expressed cellular oxidases whose products include superoxide and H_2_O_2_. XO and its precursor XOR are expressed in mammalian cells, where they localize to the cytosol and the external face of the plasma membrane [51]. Both XO and XOR can catalyze the oxidation of purines, producing superoxide radical and H_2_O_2_ [52]. Under some conditions, XO may also catalyze NO production from nitrites and nitrates, a reaction that is favoured at lower O_2_ [46]. However, the levels of these latter compounds are relatively low in culture media and this activity may therefore be of minor importance in mammalian cell culture. The K_m_(O_2_) value of bovine XO has been reported in the range of 3–6% O_2_ (Table 4). Interestingly, the relative production of superoxide versus H_2_O_2_ by XO is also sensitive to O_2_ levels within the range found in cell culture (1–21%), such that at O_2_ levels below 5% O_2_, H_2_O_2_ formation is promoted [53]. In addition, high O_2_ levels promote posttranslational modifications of XOR that decrease its specific activity [54, 55]. Thus, O_2_ levels in culture could affect the relative rates of ROS production by XO/XOR as well as the relative amounts of superoxide versus H_2_O_2_ produced.

MAO occurs as two isoforms, MAO-A and MAO-B. Both are widely distributed in mammalian tissues [56]. They localize to the outer mitochondrial membrane where they catalyze the degradation of biogenic and dietary monoamines such as norepinephrine, dopamine, tyramine, serotonin, phenylethylamine, and benzylamine in a reaction producing H_2_O_2_. The reported K_m_(O_2_) values of both isoforms vary widely (Table 4) due to a complicated reaction mechanism in which O_2_ affinity is strongly affected by monoamine concentrations (reviewed in [57]). MAO enzyme activities are nonetheless predicted to be sensitive to O_2_ over the range of 5–18%. Because the H_2_O_2_ produced by MAOs is near the mitochondrial compartment, aberrant mitochondrial and cytosolic redox signaling and/or macromolecular damage may be caused by MAO enzymes in the O_2_ conditions typical of standard cell culture.

### 1.8. Oxygenases

Oxygenases catalyze the incorporation of oxygen into an organic substrate. Heme oxygenase (HOX), lipoxygenase (LOX), and cyclooxygenase (COX) are all widely expressed in mammalian tissues and cell lines. HOX is important in the process of heme degradation, while both COX and LOX assist in the breakdown of arachidonic acid via two separate pathways.

HOX is localized in the endoplasmic reticulum (reviewed in [58]). There are two isoforms, HOX-1 and HOX-2, sharing an amino acid sequence identity of >45%. While HOX-2 is constitutively expressed, HOX-1 is induced by endogenous and exogenous stressors such as heavy metals, pharmacological agents, inflammatory mediators, UV light, and oxidative stress. HOX enzymes oxidize heme to produce carbon monoxide, Fe^2+^, and biliverdin. HOX purified from chicken liver has maximal activity at O_2_ levels as low as 1.5% ([59]; Table 4), indicating that this enzyme is likely O_2_ saturated even in cells cultured in physioxia.

COX is a dioxygenase that catalyzes the first step of arachidonic acid or linoleic acid breakdown leading to the production of prostanoid derivatives (reviewed in [60]). COX enzymes modulate cell growth and signaling pathways and are implicated in cancer progression [61]. There are two isoforms, COX-1 and COX-2, with an amino acid sequence homology of ~60%. COX enzymes localize to the endoplasmic reticulum membrane and nuclear envelope. These enzymes catalyze two-step reactions. In the first step, cyclooxygenase activity using molecular oxygen produces the hydroperoxide prostaglandin G_2_ intermediate. Peroxidase activity is then observed resulting in the formation of prostaglandin H_2_. The K_m_(O_2_) value of COX-1-catalyzed arachidonic acid oxidation varies between 0.4% and 3.1% O2, while the K_m_(O_2_) value of COX-2 is somewhat lower (Table 4). Activities of COX-1 and COX-2 saturate at around 10–20% O_2_ [62, 63]. Thus, these enzymes are also O_2_ sensitive in the range of interest, and as their products modulate various cellular activities including cell growth and differentiation [61], it is possible that their increased activities at 18% O_2_ affect studies of cell physiology in culture.

LOX catalyzes the deoxygenation of polyunsaturated fatty acids, producing a variety of oxygen and lipid radicals under some conditions (reviewed in [60]). There are six isoforms of LOX that are widely distributed in mammalian tissues and cells. LOX enzymes produce ROS and lipid radical species that participate in intracellular signaling pathways. O_2_ levels affect the lipoxygenase reaction in complex ways [64, 65], but the measured K_m_(O_2_) values are 1–2.6% O_2_ (Table 4), making reaction rates O_2_ sensitive in the relevant range.

### 1.9. Transcriptional Regulation of ROS/RNS-Producing Enzymes

In addition to acute effects on the activities of enzyme-catalyzed reactions, O_2_ levels in cell culture may affect the expression of various O_2_-consuming enzymes and organelles. HIF-1 and HIF-2 are heterodimeric transcription factors whose activities are regulated by O_2_ via the degradation of the *α*-subunits (HIF-1*α*, HIF-2*α*). This reaction is catalyzed by prolyl hydroxylase (PHD), which uses O_2_ and 2-oxoglutarate to hydroxylate the HIF-*α* subunits leading to their subsequent ubiquitination and degradation. A second reaction, hydroxylation of an asparagine in HIF-1*α* catalyzed by the factor inhibiting HIF-1 (FIH), inhibits HIF-1 transcriptional activity. Together, O_2_ regulates the levels and activity of HIF-1 and/or HIF-2. Both hydroxylation reactions are O_2_ sensitive within the physioxia to 18% O_2_ range. Mammalian PHD isoforms have a K_m_(O_2_) value~25% O_2_ and FIH K_m_(O_2_) value~11% (Table 4). In addition, PHD activities may be modulated by ROS/RNS produced by many of the enzymes discussed above at higher rates under standard culture conditions.

**Figure 3:**
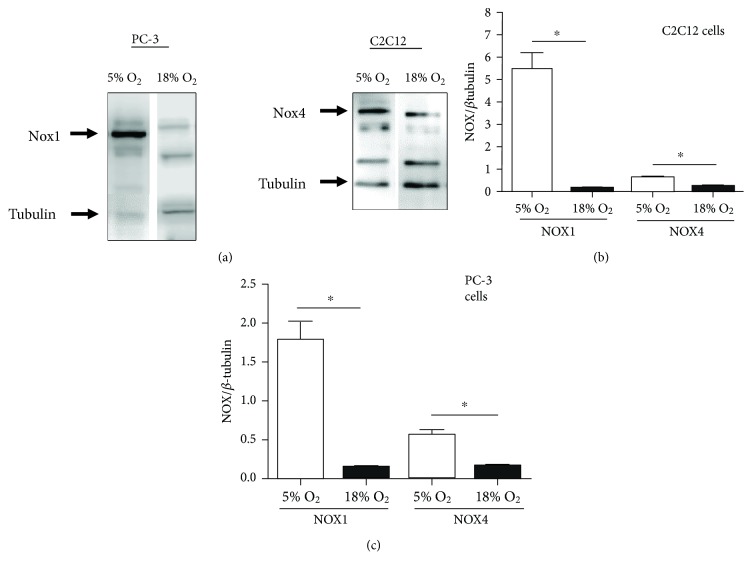
Reduced levels of NADPH oxidases 1 and 4 at 18% versus 5% O_2_. (a) Representative Western blots showing Nox1 and *β*-tubulin in PC-3 cells or Nox4 and *β*-tubulin in C2C12 cells, at 5% and 18% O_2_. (b, c) Average Nox1 signal (b) or Nox4 signal (c) standardized to *β*-tubulin. Total cellular proteins were extracted by treating cells with NP-40 buffer (150 mM NaCl, 1% NP-40, 50 mM Tris-HCl pH 8.0). Total protein (15 *μ*g per sample) was resolved on 10% SDS-PAGE, transferred to a polyvinylidene difluoride membrane, and probed for Nox1 or Nox4. *β*-Tubulin was used as an internal loading and transfer control. All antibodies were purchased from Novus Biologicals; Nox1 (NBP1-31546), Nox4 (NB110-5885), *β*-tubulin (NB600-936). Data were analysed using two-tailed Student's *t*-tests. Bars represent means ± SEM from at least five independent experiments. ^∗^*p* < 0.05. The identities of bands at intermediate molecular weight are unknown.

Hundreds of genes are transcriptionally regulated by HIFs, and interestingly, most of the ROS/RNS-producing enzymes discussed above are amongst them. In our experiments, the relative levels of Nox1 and Nox4 are much higher at 5% compared to 18% O_2_ (Figure 3). Indeed, Nox4, all three NOS isoforms, LOX, COX, MAO, and HOX are all transcriptional targets of HIFs (Table 5). In addition, several AQPs are HIF regulated, indicating that the H_2_O_2_ permeability of cellular membranes is likely different at physioxia versus 18% O_2_. It is important to note that virtually, all data regarding the HIF regulation of these enzymes has been collected using 18% O_2_ as “normoxia” and 1% O_2_ as hypoxia but similar results are expected for 5% versus 18% comparison.

In terms of the impact on cell culture experiments, the effects of O_2_ on the specific activities (based on K_m_(O_2_) values) versus levels of O_2_-consuming proteins (based on transcriptional upregulation) would tend to oppose each other, though our measurement of higher rates of cellular ROS production at 18% O_2_ versus 5% O_2_ suggests that they do not cancel. It is unclear to what extent the combined effects of media O_2_ on acute metabolic flux through the various O_2_-consuming pathways and the transcriptional regulation via HIF-1/2 activation of their component proteins, would impact cell physiology. Indeed, it seems impossible to predict how the hyperoxia of cell culture will impact the numerous O_2_-sensitive, O_2_-consuming, metabolic reactions overall given these concomitant changes in specific activity and expression.

### 1.10. Conclusions

Under standard cell culture conditions, media O_2_ levels of typically~18% are hyperoxic with respect to the 1–6% experienced by most mammalian cells *in vivo* (Table 1). This disparity has important consequences for the accurate modeling of *in vivo* cell physiology, so it is important to have a comprehensive understanding of how O_2_ affects specific ROS/RNS-producing processes. Although rates of ROS production from mitochondrial respiration are relatively insensitive to increases in O_2_ levels from 5% to 18% O_2_, many widespread O_2_-consuming cellular enzymes are very sensitive in this same range. In particular, the activities of Nox4, nNOS, eNOS, and both MAO isoforms appear to be strongly induced at higher O_2_ levels. These enzymes will thus produce substantially more ROS/RNS, potentially affecting the states of intracellular signaling pathways and the downstream events they are regulating. This is unlikely to be an appropriate starting point upon which to impose further stresses and expect a “normal” physiological response that mimics an *in vivo* context.

In addition to acute effects of O_2_ on flux through specific metabolic pathways, there is evidence that chronic exposure to the hyperoxia of cell culture will also affect the expression of Nox, NOS, MAO, and other O_2_-consuming enzymes (Table 5). Presumably, this is a homeostatic mechanism for tuning these reactions to O_2_ availability. However, it again establishes a baseline condition that may not accurately model the *in vivo* state. Even the permeability of cellular membranes to H_2_O_2_, NO, or O_2_ may be influenced by O_2_-mediated transcriptional regulation of AQPs, given their role in facilitating the diffusion of these molecules (Table 5).

The specific effects of O_2_ on cellular ROS/RNS production described above point to the importance of culturing cells at physiologically relevant O_2_ levels. In most cases, this requires lowering the incubator headspace O_2_ levels. However, this can cause pericellular and intracellular hypoxia, depending upon cell density and mitochondrial respiration rates. Therefore, it is further necessary to monitor pericellular O_2_ levels and take steps to maintain them within a physiologically relevant range. We have found that standing O_2_ gradients from the top of the media column to the pericellular region are present in undisturbed cell culture and that regular gentle mixing (e.g., via a rocker plate) can abolish these. Our simple solution to this problem will be presented in a future publication.

## Figures and Tables

**Table 1:** Average oxygen levels measured in human tissues and cells *in vivo*.

Tissue/compartment	pO_2_ (%)	Reference
Brain	4.4 ± 0.3	[66]
Brain glioma cells (intracellular)	4.5 ± 0.5	
Skin	1.1–4.6	[66, 67]
Liver	5.4 ± 0.7	[66, 68]
Liver (mitochondrial)	3-4	[69]
Skeletal muscle	3.8 ± 0.2	[70]
Skeletal muscle (intracellular)	3.4–4.8	Lanza et al. 2010
Kidney	9.5 ± 2.6	[66]
Kidney (intracellular)	6.6–7.9	[71]
Bone marrow	1.3–2	[72, 73]

**Table 2:** Intracellular O_2_ levels under various cell culture conditions.

Cell line and conditions	Extracellular O_2_ (%)	Intracellular O_2_ (%)	References
Confluent, differentiated PC12 cell monolayer	20	~15	[74]
	10	~7	
	6	~1.5	
HeLa	20	~17	[75]
	10	8-9	
	5	2–5	
Undifferentiated PC12 cells	20	~15	[76]
	8	1-2	
Mouse embryonic fibroblasts	20	6–8	[76]
Mouse embryonic fibroblasts	20	~14	[9]
	9	2-3	
	5	~0.5	

**Table 3:** K_m_(O_2_) and V_max_ values for H_2_O_2_ production from isolated liver mitochondria respiring on various substrates.

Substrates	K_m_(O_2_) (%)	H_2_O_2_ production (pmol min^−1^·mg protein^−1^)
Glutamate + malate with malonate	0.025	250
Succinate (− rotenone)	0.179	330
Succinate (+ rotenone)	0.070	105
Glutamate, malate, and succinate	0.050	330
Palmitoylcarnitine (− rotenone)	0.100	290
Palmitoylcarnitine (+ rotenone)	0.398	250

*Sites*		
Complex III Q_o_ site	0.199	150
Complex I FMN site	0.019	170
Complex I electron backflow	0.090	135
ETFQOR	0.498	200

All values are from Hoffman et al. 2009 and measured using an Amplex Red/horseradish peroxidase assay in the presence of superoxide dismutase at 37°C.

**Table 4:** K_m_(O_2_) values for various O_2_-consuming enzymes.

Enzyme	Product	K_m_(O_2_) (%)	Details	Reference
Nox2	O_2_^−^	3.5		[77]
Nox2	O_2_^−^	3.1		[78]
Nox2	O_2_^−^	2-3		[19]
Nox2	O_2_^−^	2-3		[79]
Nox4	H_2_O_2_	18		[78]
Nox4	H_2_O_2_	18		[19]
Nox4	H_2_O_2_	16–20		[79]
nNOS	NO	2.3	Purified bovine enzyme	[45]
nNOS	NO/O_2_^−^	2.2	Partially uncoupled rat enzyme	[42, 43]
	O_2_^−^	3.4	Fully uncoupled rat enzyme	
nNOS	NO	15.7	Purified rat enzyme	[47]
nNOS	NO	39.8	Purified rat enzyme	[80]
nNOS	NO	28.5	Kinetic model based on rat enzyme data	[81, 82]
eNOS	NO	0.8	Purified bovine enzyme	[45]
eNOS	NO	0.3	Kinetic model based on rat enzyme data	[82, 83]
iNOS	NO	0.63 ± 0.09	Purified bovine enzyme	[45]
iNOS	NO	11.0	Isolated enzyme assay	[84]
iNOS	NO	10.6	Kinetic model	[82]
NOS	NO	3.1–10.8	Unspecified isoform	[48, 85]
XO	O_2_^·−^/H_2_O_2_	2.2–6.8	Isolated from bovine milk	Fridovich et al. 1962, 1964
MAO	H_2_O_2_	3.4–28	Mammalian enzymes; various substrates	[86–88]; reviewed in [57]
HO	CO, iron, and bilirubin	<1.5	Unspecified isoform from chicken liver	[59]
LOX	H_2_O_2_	1–2.6	Unspecified isoform	[65]
COX-1	Various	0.4–3.1	Arachidonic acid substrate	[63, 89–91]
COX-2	Various	1.3–1.5	Arachidonic acid substrate	[63, 65]
PHD	Modified HIF	41–46	Various substrates	[92]
FIH	Modified HIF	4–12	Various substrates	[92]

Nox: NADPH oxidase; NOS: nitric oxide synthase; XO: xanthine oxidase; MAO: monoamine oxidase; HO: heme oxygenase; LOX: lipoxygenase; COX: cyclooxygenase; PHD: prolyl hydroxylase; FIH: factor inhibiting HIF-1.

**Table 5:** Hypoxia effects on the expression of some O_2_-consuming enzymes.

Enzyme	Effect	Reference
Nox1	Transcriptional stimulation by HIF-1	Goyal et al. 2004
Nox4	Transcriptional stimulation	[93]
nNOS	Transcriptional stimulation	[94]
eNOS	Transcriptional stimulation	[95]
	Stabilization of mRNA	[96]
iNOS	Transcriptional stimulation by HIF-1	[97–99]
XO	Transcriptional stimulation	[54]
MAO	Transcriptional stimulation	[100]
HO	Transcriptional stimulation	[101]
LOX	Transcriptional stimulation	[102]
COX	Transcriptional stimulation	Demasi et al. 2004;
AQP1	Transcriptional stimulation	[103]
AQP3	Transcriptional stimulation	Hoogewijs et al. 2016
AQP5	Transcriptional repression	[34]
AQP9	Unclear mechanism	Castro-Parodi et al. 2013

Nox: NADPH oxidase; NOS: nitric oxide synthase; XO: xanthine oxidase; MAO: monoamine oxidase; HO: heme oxygenase; LOX: lipoxygenase; COX: cyclooxygenase; AQP: aquaporin.
